# Calculation of Left Ventricular Relaxation Time Constant-Tau in Humans by Continuous-Wave Doppler

**DOI:** 10.2174/1874192400802010009

**Published:** 2008-04-25

**Authors:** Xufang Bai

**Affiliations:** Henderson Research Center, McMaster University, Hamilton, Ontario, Canada

## Abstract

Left ventricular relaxation time constant, Tau, is the best index to evaluate left ventricular diastolic function, but the measurement is only available traditionally in catheter lab. In Echo lab, several methods of non-invasive measurement of Tau have been tried since 1992, however almost all the methods are still utilizing the same formula to calculate Tau as in catheter lab, which makes them inconvenient, time-consuming and sometimes not very accurate. Based on Weiss’ formula and simplified Bernoulli’s equation, a simple method is developed by pure mathematical derivative to calculate Tau by continuous-wave Doppler in patients with mitral regurgitation.

## INTRODUCTION

Diastolic function of the left ventricle is abnormal in many cardiac diseases, and usually appears ahead of the abnormal systolic function. In 1976, Weiss *et al. *[1] found that left ventricular pressure was able to be plotted and fit to an exponential function:

(1) $$P=e^{-t/T+B}$$

where P is the left ventricular pressure, e=2.71828…, is the natural logarithmic base, t is time from –dP/dt max, T is left ventricular relaxation time constant, also called Tau, and B is a constant. From Eq. (1), the derivative of the left ventricular pressure is expressed by:

(2a) $$\mathrm{dP}=e^{-t/T+B}\left(-1/T\right)\mathrm{dt}=P\left(-1/T\right)\mathrm{dt}$$

or

(2b) $$T=P/\left(-\mathrm{dP}/\mathrm{dt}\right)$$

Since P and -dP/dt are variables that can be obtained in catheter lab, the important parameter, Tau, becomes available through a simple calculation as shown above and its accuracy can be ensured through the accessibility of P and -dP/dt in the catheter lab. Obviously this method is inconvenient and invasive. Since 1992 many methods of noninvasive measurement of Tau by continuous-wave Doppler in patients with mitral regurgitation have been reported [2-5]. However, the derivation of the important parameters seems time-consuming and complicated *via *the proposed traditional method in Echo lab. In detail, non-invasive measurement of Tau has been based on the formula: T=P/(-dP/dt). Left ventricular pressure P was calculated as the sum of the ventriculoatrial gradient and the LAP (left atrial pressure). The calculation of the ventriculoatrial gradient can be simply available to be 4v^2^, where v is an instantaneous regurgitation velocity across mitral valve, based on the simplified Bernoulli’s equation. However, the determination of LAP is more complicated, since LAP varies with time and a simultaneous calculation of Tau *via *the LAP at the exact moment for the measurement of the P and of the -dP/dt is indispensable. In view of the difficulty, an equation to calculate Tau and LAP were proposed as follows [6]:

(3) $$\mathrm{IVRT}=T\left(\mathrm{LnACP}-\mathrm{LnLAP}\right)$$

where IVRT is the isovolumic relaxation time, T is Tau, and ACP is aortic valves closure pressure. This method assumes that the maximum of -dP/dt occurs at the same time when the aortic valves close. However, the maximum of –dP/dt may not always happen exactly at the same time as aortic valves closure, and there could be a delay sometimes. [7,8] In addition, after the return of pressure to the level of end-diastolic pressure, passive viscoelastic properties may be of importance and its effect on the evaluation of Tau should be modeled. [1] In another word, neither the beginning nor the end of this period fits the function P=e^*-t/T+B*^ very accurately, although the middle part of the isovolumic diastolic period does. Therefore, the applicability of the proposed method could be inadequate under some circumstances. So a simple and accurate method seems indispensable for an efficient and effective measurement of the parameter Tau in clinic.

## DEDUCTION OF FORMULAS

In the Echo examination for patients with mitral regurgitation, the left ventricular pressure can be expressed as:

(4) P=ΔP+LAP

where ΔP is the pressure gradient between left atrium and left ventricle. Substituting Eq. (1) and the simplified Bernoulli’s equation: ΔP=4v²into Eq. (4) leads to the following equation:

(5) $$e^{-t/T+B}=\Delta P+\mathrm{LAP}=4v^{2}+\mathrm{LAP}$$

A natural logarithmic transformation on both sides of the above equation results in the expression:

(6) $$-t/T+B=\ln\left(4v^{2}+\mathrm{LAP}\right)$$

**Fig. (1):**
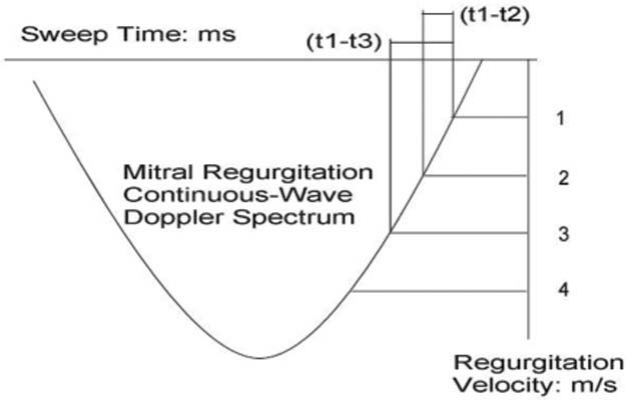
Schematic description of measurement of time intervals (t1-t3) and (t1-t2) on the descending limb of mitral regurgitation continuous-Doppler spectrum.

Three points, (t1, 1m/s), (t2, 2m/s) and (t3, 3m/s), are chosen on the descending limb of the mitral regurgitation continuous-wave Doppler velocity curve (Fig. **1**), and substituted into Eq. (6) respectively, which can come across the following three equations:

(7a) $$-\mathrm{t1}/T+B=\ln\left(4x1^{2}+\mathrm{LAP}\right)$$

(7b) $$-\mathrm{t1}/\mathrm{2T}+B=\ln\left(4x2^{2}+\mathrm{LAP}\right)$$

(7c) $$-\mathrm{t3}/T+B=\ln\left(4x3^{2}+\mathrm{LAP}\right)$$

From the difference comparison of Eqs. (7a) and (7b), we can find:

(8a) $$-\left(\mathrm{t1}-\mathrm{t2}\right)/T=\ln\left(4x1^{2}+\mathrm{LAP}\right)-\ln\left(4x2^{2}+\mathrm{LAP}\right)\mathrm{or}:\mathrm{Tau}=\left(\mathrm{t1}-\mathrm{t2}\right)/\ln\left(\left(16+\mathrm{LAP}\right)/\left(4+\mathrm{LAP}\right)\right)$$

Similarly,

(8b) $$\mathrm{Tau}=\left(\mathrm{t1}-\mathrm{t3}\right)/\ln\left(\left(36+\mathrm{LAP}\right)/\left(4+\mathrm{LAP}\right)\right)$$

From the above formulas (8a) and (8b), both Tau and LAP can be calculated after we measure two time intervals: (t1-t3) and (t1-t2). Fig. (**1**) shows how measurement is done.

## ADVANTAGES OF OUR METHOD

The deduction based on Weiss’ formula, simplified Bernoulli’s equation and mathematical derivative makes the method almost universal. For calculation of Tau and LAP based on of Eq. (8a) and (8b), a simple computer program can be developed to make the calculation easier. The method is applicable to all patients with acceptable quality of mitral regurgitation spectra only if the application of the Weiss’ formula and simplified Bernoulli’s equation are acceptable.

## DERIVATION OF LAP

**Fig. (2):**
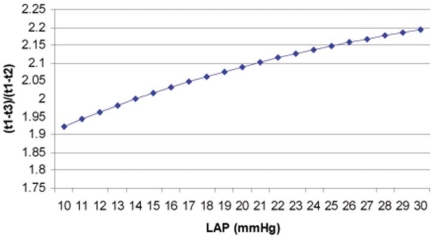
The curve derived from Eq. (9) shows the relation of (t1-t3)/(t1-t2) and LAP.

Calculation of the ratio of Eqs. (8a) and (8b) leads to the following equation:

(9) $$\left(\mathrm{t1}-\mathrm{t3}\right)/\left(\mathrm{t1}-\mathrm{t2}\right)=\ln\left(\left(36+\mathrm{LAP}\right)/\left(4+\mathrm{LAP}\right)\right)/\ln\left(\left(16+\mathrm{LAP}\right)/\left(4+\mathrm{LAP}\right)\right)$$

from this equation we can draw the conclusion that LAP is determined by the ratio of (t1-t3)/(t1-t2) on the descending limb of mitral regurgitation continuous wave Doppler spectrum. (Fig. **2**) Interestingly, when (t1-t3)/(t1-t2)=2 is considered, or t2 is in the middle of t1 and t3 in terms of time, from Eq. (9), we can find the corresponding result, LAP=14 mmHg. Further study about this relationship between (t1-t3)/(t1-t2) and LAP and must be very exciting and fruitful.

## DERIVATION OF TAU=1.2(t1-t3) FOR MOST CIRCUMSTANCES

From Eq. (8b) Tau= (t1-t3)/ln((36+LAP)/(4+LAP)), we have:

(10) $$\mathrm{Tau}/\left(\mathrm{t1}-\mathrm{t3}\right)=1/\ln\left(\left(36+\mathrm{LAP}\right)/\left(4+\mathrm{LAP}\right)\right)$$

To better understand the relation of LAP and Tau/(t1-t3), Fig. (**3**) is plotted based on Eq. (10).

Further in detail, from Eq. (10), we have the following data (Table **1**), showing when LAP is around 19-22 mmHg, Tau/(t1-t3) is around 1.2, or Tau = 1.2 (t1-t3).

It is seen that when LAP is around 19-22 mmHg, Tau/(t1-t3) is approximately to be 1.2. This could be true for most of the situations, because of the following aspects:

(a). Normal LAP is around 5-15 mmHg. It reaches its maximum just after aortic valve closes and isovolumic diastolic period starts. Shortly after this period, we have the maximum of –dP/dt and will measure the LAP for the calculation of Tau. When mitral regurgitation happens, there should be a surge of the pressure within the left atrium, which will make the LAP more likely to be around 19-22 mmHg. Another consideration is in normal heart LAP begins to decrease after aortic valve closes. [2] Once there is mitral regurgitation, LAP can keep going up until the end of isovolumic relaxation period. Both the decrease and the increase trends can correct each other to some degree. In the mean time, the four pulmonary veins connected with the left atrium can buffer some pressure surge within the left atrium. All these considerations make 19-22 mmHg a good guess for LAP in the middle the isovolumic relaxation period.

(b). Nishimura *et al. *[3] optimized 5 methods of calculation of Tau with different LAPs. They concluded that knowledge of the left ventricular end-diastolic pressure was best, which was 22±10.2 mmHg. They also suggested a less optimal but acceptable method of assuming LAP to be 20 mmHg. From Table **1**, we know either of these guesses of LAP shows that Tau/(t1-t3) is around 1.2. Interestingly, Nishimura *et al. *[3] also got a formula by observing the data collected: (t1-t3)=0.8Tau+13. This formula can be rewritten like this: Tau=((t1-t3)-13)/0.8=1.25(t1-t3)-10.4, which is exactly very similar with our simplified formula: Tau=1.2(t1-t3). The latter is purely a product of mathematics deduction.

## LIMITATIONS

Only data for patients with mitral regurgitation and fairly good quality of Doppler spectra can be used to calculate Tau. Therefore, for most the patients in clinic, the noninvasive measurement of Tau and LAP is still inapplicable.

## FUTURE ENDEAVOR

When we tried to calculate LAP from (t1-t3) and (t1-t2), we presumed the LAPs were the same at the 3 time points: 3m/s, 2m/s and 1m/s. In fact, some difference does exist among the 3 time points. If more precise measurement methods are available, it is possible to choose the 3 points more closely, such as 2.1m/s, 2m/s and 1.9m/s, which is helpful to decrease the systematic error caused by LAP variation.

The development of digital techniques with more pixels will give us more information and a faster sweep speed, for example, 200 mm/s or more will make the measurement more accurate.

In this digital era, the spectra border has already been digitized, we can expect integration of these formulas (8a) and (8b) to an Echo machine will enable a simple and effective derivation of Tau and LAP immediately after we get a mitral regurgitation continuous-wave Doppler spectrum.

Further investigations with large sample number from primary patient data will be helpful to fully prove the method.

## Figures and Tables

**Fig. (3):**
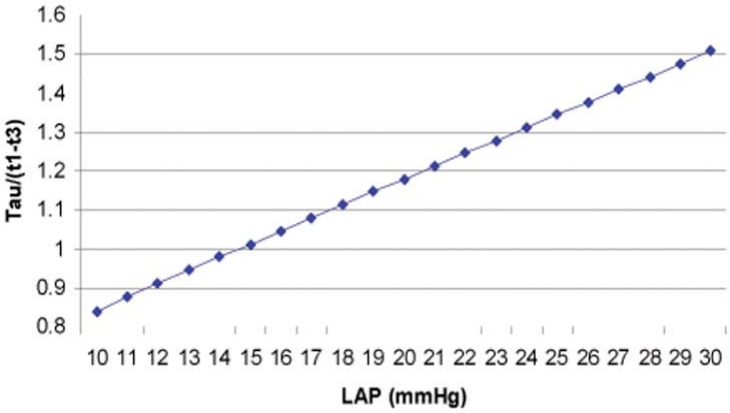
The curve derived from Eq. (10) shows the relation of Tau/(t1-t3) and LAP.

**Table 1.** Some Data Derived from Eq. (10)

**LAP (mmHg)**	**Tau/(t1-t3)**
17	1.08
18	1.11
19	1.15
20	1.18
21	1.21
22	1.25
23	1.28
24	1.31
